# Factors Influencing Eating Habits of Video Gamers and Professional eSports Gamers in Peru

**DOI:** 10.3390/foods14213597

**Published:** 2025-10-22

**Authors:** Jimena Mujica Caycho, Michelle Lozada-Urbano, Rubén Aguirre-Ipenza, Pavel J. Contreras

**Affiliations:** 1 Programa académico de Nutrición y Dietética, Universidad Privada Norbert Wiener, Lima 15001, Peru; mujicajimenac@gmail.com (J.M.C.); pavel.contreras@uwiener.edu.pe (P.J.C.); 2 Faculty of Health Sciences, Universidad Continental, Lima 15046, Peru; raguirrei@continental.edu.pe

**Keywords:** video gamers, eating habits, gamers, streamers, eSports

## Abstract

eSports and recreational video gaming are expanding in Peru, yet evidence on gamers’ dietary habits and correlates is scarce. We aimed to identify factors associated with eating habits among Peruvian video gamers and professional eSports players. Quantitative and cross-sectional study (Peru, 2023). A culturally adapted version of the German Sport University Cologne questionnaire (28 items; Cronbach’s α = 0.86) was administered online using non-probability snowball sampling. The primary outcome was eating-habit classification (adequate vs. inadequate) based on the instrument’s scoring. Associations with hypothesized correlates (e.g., gaming-related influences, peer interaction, advertising) were assessed with χ^2^ or Fisher’s exact test (α = 0.05). We analyzed 288 respondents (median age 21 years). Overall, 77.8% exhibited inadequate eating habits. Daily water intake was reported by 72%, whereas daily fruit and vegetable consumption was 21% and 32%, respectively. Peer interaction within the gaming environment (*p* = 0.037) and the perceived influence of video games (*p* = 0.031) were significantly associated with poorer eating habits. Sitting time, number of meals per day, daily water intake volume, and weekly gaming hours showed no significant association (all *p* > 0.05). Most Peruvian gamers report suboptimal diets. Social dynamics in the gamer community and gaming-related influences are linked to poorer eating habits, suggesting that nutrition strategies should be embedded in gamer ecosystems (teams, communities, platforms). Longitudinal and interventional studies are warranted to test targeted behavior-change approaches.

## 1. Introduction

A video gamer is a person who enjoys playing video games and spends a lot of time playing them [1]. In Peru, the video game industry is growing exponentially, both because of competitive element and the significant prizes offered [2]. Thus, we have a growing population of gamers [3] who play for several hours [4], which encourages professionalism in this activity [5,6]. Video games are associated with sedentary lifestyles, which, in turn, are associated with weight gain and other health risks [7,8]. Therefore, eSports players may require medical consultation and nutritional counseling [9] to optimize their health and performance.

There is a significant gap in the research on the nutrition of professional eSports competitors and amateur video gamers [10]. The skills of a video gamer, such as eye-hand response and immediate strategic capacity, depend in part on an adequate intake of micronutrients such as magnesium, phosphorus, potassium, sodium, and B vitamins, among other substances [11,12]. For authors such as Kim, water intake was found to be important to improve gamers’ practice, and it was found to be necessary to improve the quality of the diet they receive for training and playing, especially in young adults [13].

In Germany, a study of 1066 amateur and professional video game players found that 95% reported being in good or excellent health, and both groups showed an average consumption of 2.7 ± 1.8 servings of fruit and vegetables per day [14]. Another study (2021) in the same country evaluated 820 video gamers of different performance levels and found an intake of red meat, sausages, and sugary drinks above the recommended intake [15].

A video gamer is a person who enjoys playing video games and spends a lot of time playing them [16]. Also known as a “gamer,” the designation refers to someone who is interested in playing, mastering, and learning about video games, often with years of experience [17]. A professional eSports player is someone who competes at the highest level of a video game and is able to generate money to cover their basic expenses through eSports [18]. The term eSports refers to a competitive environment for a particular sector of video games in which there is an organization involving leagues and tournaments, media outlets, etc., and a community of fans [19]. Some of the most widespread games are MOBAs (multiplayer online battle arenas) [20] such as DOTA2. There is evidence documenting long hours of screen exposure among these players, often accompanied by sedentary behavior and unhealthy dietary patterns, which have been linked to an increased risk of chronic diseases [21].

## 2. Materials and Methods

This study was conducted using a quantitative and cross-sectional design, selected for its suitability in identifying associations between key variables at a single point in time. Cross-sectional studies are particularly useful for estimating the prevalence of specific behaviors and their relationship with health outcomes, without requiring long-term follow-up [22]. This design is widely applied in public health and social sciences research because of its efficiency, relatively low cost, and ability to generate hypotheses for future longitudinal studies [23,24].

### 2.1. Population, Sample, and Sampling

The population consisted of video gamers and eSports gamers of both sexes of Peruvian nationality. In total, 288 video gamers participated. The sampling methods were non-probabilistic, purposive, and chain. This allowed for the inclusion of video gamers at the professional performance level and former professional gamers, who are fewer in number and less accessible.

### 2.2. Selection Criteria

The inclusion criteria were both sexes between 18 and 40 years of age, residing in Peru during the survey. For professional eSports gamers, criteria included belonging to a professional organization and playing video games for a minimum of 35 h per week (which could be 5 h per day). For video gamers, criteria included playing more than once a week but not participating in tournaments and official leagues. Before starting the questionnaire, a filter question about the country of residence was shown. If their residence was outside Peru, the questionnaire ended, and the participant was excluded.

### 2.3. Collection Techniques and Instruments

The instrument was distributed online through different Peruvian streamer communities, in video game forums, and by personal contact to Peruvian eSports organizations and teams that helped to disseminate the instrument.

In this research, the survey technique was used. The questionnaire was prepared in Google Forms based on the questionnaire from the German Sport University of Cologne and sent virtually, with additional questions covering sociodemographic aspects.

The questionnaire was created in 2018 by the Institute for Movement Therapy and Movement-Oriented Prevention and Rehabilitation of the German Sport University Cologne in the eSports project. It was created via the online survey instrument Unipark (Questback GmbH, Cologne, Germany). The ethics committee of the German Sport University Cologne approved this study (reference: 053/2018). The instrument, which assesses the frequency of food consumption, was kindly provided by the researchers who devised it in the German language. The instrument has 28 items divided according to (a) general data, (b) eating habits, and (c) perception of factors influencing food consumption.

For the cut-off points that classified participants into those with adequate or inadequate eating habits, the scoring is as follows: good eating habits: ≥112 points; bad eating habits: <112 points.

The main variables were eating habits, time spent playing video games, advertising, interaction with friends or peers, video games, weekly gaming hours, and time spent sitting or resting.

Age was categorized into three segments based on decades of life, thus including those under 20, those aged 20 to 30, and those over 30.

Factors influencing food consumption we as follows:

Influence of the family environment (food preparation, foods that meet cultural preferences): [The family environment does have an influence: ≥4 points in question 23]; sociocultural influence (sponsors, advertisements, social rules of video games): [Behavior does influence video games: 10–13 points (questions 11, 24, and 27)]; video game behavior (hours of play, screen time, physical activity): [Yes, the socio-cultural environment does have an influence: 8–10 points (questions 25 and 26)].

### 2.4. Validation of the Instrument

The questionnaire received in German was translated by a certified translator (No. 0007-2023; CTP No. 0968). To validate the instrument, it was necessary to culturally adapt some items and their alternatives to our target population to avoid confusion during its application, as in the case of items 4 and 5 (“Indicate the highest academic degree you hold” and “What is your current professional situation?”), as well as to add items that allow for the desired results to be obtained in accordance with the objectives of this study. The questionnaire was submitted for content validation by expert university lecturers who teach sports nutrition. Subsequently, a pilot test was carried out with a small sample of 30 participants who were online gamers, and the results of the analysis confirmed the suitability and relevance of the instrument.

Internal consistency reliability analysis was undertaken for the questionnaire developed by the Institute for Movement Therapy and Movement-Oriented Prevention and Rehabilitation at the German Sport University in Cologne. The Test scale had a total of 28 items in the scale and an average inter-item covariance equal to 0.37004. To demonstrate that the instrument used in this research has the psychometric property of reliability, the internal consistency analysis method was applied using STATA 18 software, resulting in a reliability coefficient of 0.8586, which is classified as good.

The adaptation of questionnaires to the cultural context of the target population is essential to ensure their validity and reliability. Several authors emphasize that direct translation is insufficient as linguistic, social, and cultural differences can alter the interpretation of the items and compromise data comparability [25,26]. For this reason, we introduced specific modifications to the original instrument to align the terminology and examples with the local context, thereby improving its comprehensibility and relevance for respondents. This approach follows established guidelines for cross-cultural adaptation of self-report measures, which recommend considering semantic, idiomatic, experiential, and conceptual equivalence [27].

Expert judgment was obtained from three specialists in nutrition and public health, who reviewed the translated instrument and this questionnaire and independently rated each item for clarity, cultural adequacy, and relevance.

### 2.5. Data Processing and Analysis

The database was validated and edited to detect implausible data. The data obtained were then coded and tabulated in a Microsoft Excel file for subsequent analysis using the statistical program STATA version 18.0.

Descriptive statistics were performed, generating frequencies and percentages for categorical variables, as well as medians and interquartile ranges for numerical variables, given that their distributions were non-normal. The relationship between variables was evaluated using the chi-square statistic or Fisher’s exact test when the statistical assumptions of chi-square were not met. The results of the association analysis are shown in double-entry tables.

For all analyses, a *p*-value < 0.05 and a confidence level of 95% were considered for the statistical decision.

## 3. Results

A total of 288 participants, mostly young males, were included in this study. Seven additional respondents were excluded because they did not complete the survey.

### 3.1. Demographic and Food Intake

A total of 94.7% (273) of the participants were male. The average time spent playing weekly was 21 h, and the average times spent sitting or without physical activity were 8 h or more, i.e., 36.5% (105), or less than 8 h, i.e., 63.5% (183). Participants mainly have a high school-level education (44.8% (129)), with 4.2% (12) possessing a university degree, and about one third identified themselves as professional eSports gamers (see Table 1).

#### Food Frequency

**Figure 1:**
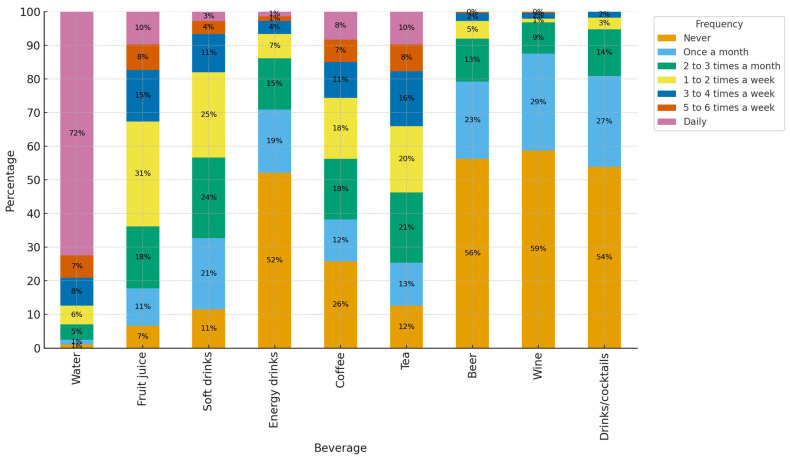
Frequency of beverage consumption.

The frequency of solid and liquid food consumption is presented in Figure 1. A total of 72% of participants reported daily water intake, while 25.3% consumed non-alcoholic beverages one to two times per week. Regarding energy drinks, 52% (N= 150) reported never consuming them, whereas 25% consumed soft drinks at least once or twice per week. Coffee consumption was less frequent, with 11% reporting intake three to four times per week and 8.3% consuming it daily.

**Figure 2:**
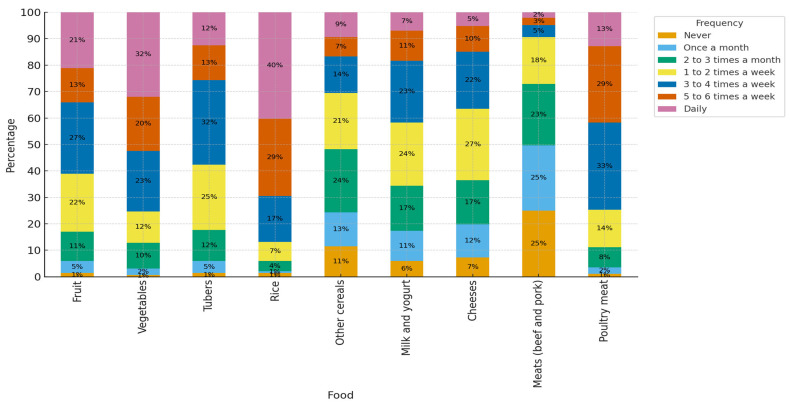
Frequency of solid food consumption.

Solid food consumption patterns are shown in Figure 2. Daily consumption of fruits and vegetables was reported by 21% (n = 61) and 32% (n = 92), respectively, while rice was consumed daily by 40% (n = 116). Milk and yogurt intake ranged from one to two times per week to three to four times per week. More than half of the participants consumed beef between three to six times per week. Poultry meat consumption three to four times per week was reported by 33% (n = 95). Processed meats, such as ham or sausages, were consumed by 26% of participants two to three times per month, while the same percentage (26%, n = 76) reported nut consumption at a similar frequency.

The relationship between dietary habits and associated variables is summarized in Table 2. Of the total participants, 64 were classified as having adequate eating habits and 224 as inadequate. No significant associations were found between eating habits and time spent sitting, number of meals per day, daily water intake, or hours of weekly play. Among those with inadequate eating habits, 77.8% (n = 224) reported sitting less than eight hours per day, 78.9% (n = 97) consumed three meals per day, 74% (n = 97) reported drinking one to three glasses of water daily, and 75% (n = 135) played fewer weekly hours.

Significant associations were observed between eating habits and peer interaction within the gaming environment (*p* = 0.031), as well as between eating habits and the influence of video games (*p* = 0.031). In addition, 45% (n = 131) of respondents agreed that family traditions influence their current eating habits, while 27% strongly agreed. Among those classified with inadequate eating habits, 38% acknowledged the influence of advertising in the gaming environment on their eating patterns. Conversely, 51% of participants with adequate eating habits reported neither agreement nor disagreement regarding this influence (Table 3).

When ranking beverages by weekly consumption or higher (≥1–2/week), water is the most commonly consumed beverage, followed by juices and tea. Coffee maintains an intermediate level of regularity. Soft drink consumption is moderate, while energy drinks and alcoholic beverages (beer, cocktails, wine) are characterized by low frequency of intake (predominance of ‘never’/’monthly’ categories).

This figure describes the most commonly consumed beverages, measured using the frequency of consumption instrument. Alcoholic beverages and energy drinks are consumed sporadically, while tea and coffee are consumed more often during the week. More than three quarters of the population consume water daily.

It describes that the basis of the diet consists of rice and vegetables/tubers/fruit consumed on a regular basis; It notes that chicken is the most frequently consumed animal protein, while red meat is consumed only occasionally; For dairy products, it indicates widespread weekly consumption, with little daily consumption.

This figure shows the consumption of solid foods such as meat, dairy products and derivatives, cereals, rice, tubers, vegetables, and fruit. They were measured using the frequency of consumption instrument. Chicken is consumed more frequently than red meat (beef and pork). Consumption of solid foods such as meat, dairy products, and derivatives, some foods such as cereals, rice, tubers, milk, and yogurt is more frequent than cheese. Vegetables are consumed more than fruit. The alternatives were as follows: 1—never; 2—once a month; 3—two to three times a month; 4—one to two times a week; 5—three to four times a week; 6—five to six times a week; 7—daily.

**Figure 3:**
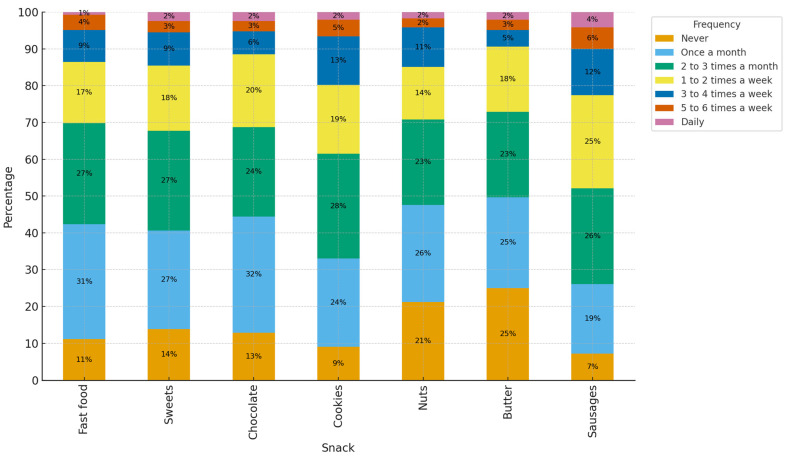
Frequency of food consumption—fast food and others.

Figure 3 shows that packaged foods, sweets, and fast food are not consumed as frequently. Just over half of the participants consume these products at least once or twice a week.

The Figure 3, show General pattern: consumption of most foods is concentrated in “once a month” and “two to three times a month”. Daily consumption is rare for all items. Never consumed: this is notably high for butter and nuts, followed by chocolate and sweets. For cold meats and biscuits, “never” is relatively low. Once a month (modal peak): almost all foods show their highest accumulation here; fast food and chocolate stand out as the most reported “once a month”. 2–3 times a month: also concentrates many cases for biscuits, sweets, chocolate, nuts, butter and cold meats (the latter with high figures). 1–2 times per week: more regular consumption of biscuits and cold meats begins to be seen; sweets and butter are also present, but to a lesser extent. 3–4 times per week: only biscuits and cold meats maintain significant quantities; fast food and chocolate/sweets drop sharply (as expected). 5–6 times per week/daily: very few cases in all foods (practically marginal).

## 4. Discussion

The findings of this research show that over one third of the participants reported sitting for more than eight hours per day, while the majority spent fewer than 29 h per week playing video games.

With respect to dietary patterns, daily water consumption was the most frequent habit, accompanied by occasional intake of soft drinks, energy drinks, and alcoholic beverages. Meat consumption was generally low, with chicken being the preferred option. Fewer than one third of participants reported daily consumption of fruits and vegetables, and a reduced intake of milk and yogurt was also observed.

Overall, this population has been frequently criticized for exhibiting unbalanced dietary habits and health-related behaviors, which appear to diverge considerably from those observed in the general population.

### 4.1. Demographic Aspects

Studies show that approximately 55% of gamers worldwide are male [28]. The predominant gender among video game gamers in Peru is male, reflecting a global trend in the gaming community. Author Garcés et al. described serious gambling problems among male university students in Peru [29].

One third of those interviewed spend more than 8 h a day sitting down. The average gaming time of professionals (36 h per week) may be related to the time they spend in order to earn a living from video games [30]. The average time spent gaming in the USA is typically around 6–7 h per week [31], whereas Latin American gamers exceed this average [32], particularly among younger men [31]. An analysis covering countries in Europe and the US showed differences, but the time spent on mobile gaming in high-GDP countries in Europe compares well with that in East Asian countries [33].

It is necessary to understand what the patterns are and what influences these patterns to improve the different gaming experiences in the region, and especially in Peru.

### 4.2. Beverage Consumption

In this study two thirds of the participants drink water daily and drink juices less frequently, one third never drink soda, and half never drink energy drinks.

The World Health Organization (WHO) recommends that adults drink between 1.5 and 2 L of water per day [34]. It is also possible to consume other beverages such as soft drinks. Compared to 14- to 17-year-olds, who drink an average of almost half a liter of soft drinks per day [35], the participants in this study clearly drink less. However, the gamers’ consumption of one serving per day is already too high and should be reduced. The high sugar content of these drinks may lead to an increased risk of diabetes, weight gain, and high blood pressure [35].

### 4.3. Energy Drinks

Energy drink brands have created strong links with the gaming community through their marketing strategies, where they advertise products as beneficial to health by increasing performance and concentration [36]. It is the adolescent and young adult groups that mainly consume these products [37]. However, the high amount of sugar in energy drinks brings with it other health risks, such as insomnia, stress, gastrointestinal discomfort [38], and habituation to a sedentary lifestyle [39]. Nevertheless, there should be an effort to achieve an overall reduction in sugar-sweetened beverages because they are associated with obesity and diseases such as diabetes [40,41,42].

Motor skills, mental agility, concentration, and processing speed are some characteristics of video gamers [39]. The psychological intensity required in this sport surpasses traditional sports, where physical capacity and effort are more important. Competition in video games poses risks to health status and behavior. Existing studies have linked participation in video games with sleep disturbances, low self-esteem and self-efficacy, anxiety, and aggression, some of which are also found among those who consume energy drinks [43,44,45].

A growing body of research has raised further concerns about the lifestyle and eating behavior of video gamers, such as a poor diet and a sedentary lifestyle [39].

### 4.4. Consumption of Fruits and Vegetables

Gamers in this study consumed different types of fruits and vegetables between three and found times a week, indicating that Peruvian gamers have inadequate consumption habits.

The WHO recommends a minimum intake of 400 g per day of these foods [38]. The position of the Academy of Nutrition and Dietetics is that in order to reduce the risk of chronic non-communicable diseases such as type 2 diabetes, cerebrovascular disease, and some types of cancer, a minimum fiber intake of 25 g/day for women and 38 g/day for men should be ensured. Likewise, ensuring this minimum intake of total fiber could increase the consumption of foods low in saturated fats, trans fats, sodium, and added sugars [38].

Studies on eSports gamers in Portugal and Brazil showed a low consumption of fruits and vegetables and a high consumption of fast food, red and processed meat, soft drinks, and caffeine-based dietary supplements, concluding that they follow an unbalanced diet [46]. Other researchers, such as Moore and collaborators, working with video gamers, identified a lower consumption of fruits and vegetables, as well as a higher consumption of sodium and saturated fats [47].

Meat consumption in Peru is among the lowest in the region, according to the National Institute of Health, with an average weekly consumption of 1.2 pieces [48]. In the present study, one fourth consume meat three to four times per week. There is evidence about the association between the development of colorectal cancer and meat products, especially processed meats, so avoiding or restricting the consumption of this food would be beneficial in the long term [49]. Fifty-three percent of the Peruvian population consumes chicken meat, thus having a greater preference in relation to beef [50].

A Peruvian study in the year 2023 mentions an increase in chicken meat consumption and is correlated with the educational level [51]. In a study conducted by Bickman, P., Tholl, C. et al. in the year 2021, the authors observed a consumption of almost 7 servings of sausages per week, 2.6 servings of chicken per week, 3.5 steaks per week, and 2.7 servings of rice among video gamers.

In Peru, the consumption of red meat, which includes certain types of sausages, has decreased [52].However, in men, there is a tendency to consume red and processed meats, while women prefer cooked vegetables [53].

The results of our study indicate an average consumption of one serving of sausages per week and approximately seven servings of rice per week, as reported by the majority of participants.

### 4.5. Influence Related to Food Consumption Habits

Among the group with inadequate consumption habits, 78% agree that time spent playing video games and advertising in the gaming environment influence their eating habits. Likewise, a quarter of the evaluated population disagreed that they were not influenced by their friends or people related to their gamer environment; however, a third of them did report feeling the influence of gamer advertising when choosing and maintaining eating habits. They disagree that interaction with gamer friends and video games influences their eating habits.

In a survey which evaluated the eating behavior of German video game and eSports gamers, it was found to be similar to that of the general German population [54].

In relation to the influence that various brands have on the gamer community, we note those that are closely related to video games, as well as others that may negatively influence eating patterns.

Some gender-specific food preferences have been noted. Grzymislawska et al. (2019) indicate that men prefer fatty foods and sweet snacks during gaming sessions, often driven by pleasure rather than health considerations, while women lean towards healthy eating and body weight control, which may be influenced by social pressures [55].

Younger gamers—i.e., those younger than 20 years—snack between meals while playing, with this being an example of impulsive eating behavior. In contrast, gamers older than 30 years demonstrate healthier behaviors [56] and may prioritize balanced eating habits [57].

### 4.6. Limitations

The main limitation of this study is that the sampling was not probabilistic, so the findings cannot be generalized to the entire population of video gamers and professional eSports gamers in the country. Likewise, being a cross-sectional study, causality cannot be established, only association. Another important limitation is the lack of information on the size of the portions ingested, which makes it difficult to accurately estimate the real intake and to formulate more accurate nutritional recommendations. Finally, recruitment was focused on participants residing in Peru under non-probability sampling, which could bias the representativeness of the sample.

## 5. Conclusions

The participants were found to be participating in far-from-healthy eating patterns; however, their intake of snacks, sweets, energy drinks, and alcohol was not above the general population average, with most reporting little or no alcohol consumption.

## Figures and Tables

**Table 1:** Demographic and food intake-related characteristics of video gamers and professional eSports gamers.

Characteristics	N (%)
Age (years) *	21 (19–25)
Age (categorized)	
18–19	88 (30.6)
20–29	176 (61.1)
30–35	24 (8.3)
Sex	
Female	15 (5.2)
Male	273 (94.8)
Academic degree	
Bachelor	64 (22.2)
Title	12 (4.2)
Technician	83 (28.8)
Secondary School	129 (44.8)
Gamer category	
Professional	104 (36.1)
Recreational	184 (63.9)
Time spent sitting or resting per day	
More than 8 h	105 (36.5)
Less than 8 h	183 (63.5)
Meals a day	
1	57 (19.8)
2	108 (37.5)
3	123 (42.7)
Daily water consumption	
1 to 3 glasses	131 (45.5)
4 to 5 glasses	97 (33.7)
6 or more glasses	60 (20.8)
Weekly playtime *	21 (12–42)
playtime (categorized)	
3 to 29 h	180 (62.5)
30 to 82 h	108 (37.5)
Type of perceived healthy habit	
Adequate	224 (77.8)
Inadequate	64 (22.2)

* Median and interquartile range.

**Table 2:** Relationship of variables with “the type of perceived healthy habit.” Statistics provided via Chi2.

Variable	Eating Habits		*p*
	Inadequate (n = 224) 77.8 (%)	Adequate (n = 64) 22.2 (%)	
Time sitting or resting			0.844
8 h or more	81 (77.1)	24 (22.9)	
Less than 8 h	143 (78.1)	40 (21.9)	
Meals a day			0.928
1	44 (77.2)	13 (22.8)	
2	83 (76.9)	25 (23.1)	
3	97 (78.9)	26 (21.1)	
Daily water consumption			0.143
1 to 3 glasses	97 (74.1)	34 (25.9)	
4 to 5 glasses	82 (84.5)	15 (15.5)	
6 to more glasses	45 (75.0)	15 (25.0)	
Weekly game hours			0.143
3 to 29 h	135 (75.0)	45 (25.0)	
30 to 82 h	89 (82.4)	19 (17.6)	

**Table 3:** Perception of influence related to healthy habits with Chi2 bivariate analysis.

Variable	Eating Habits		*p*
	Inadequate (n = 224)	Adequate (n = 64)	
	77.8 (%)	22.2 (%)	
Time spent playing video games influences your diet			0.053 *
Strongly agree	22 (66.7)	11 (33.3)	
Agree	69 (78.4)	19 (21.6)	
Neither agree nor disagree	76 (74.5)	26 (25.5)	
Disagree	41 (83.7)	8 (16.3)	
Strongly disagree	16 (100.0)	0	
Gamer propaganda influences their diet			0.269
Strongly agree	20 (69.0)	9 (31.0)	
Agree	78 (78.8)	21 (21.2)	
Neither agree nor disagree	70 (73.7)	25 (26.3)	
Disagree	37 (88.1)	5 (11.9)	
Strongly disagree	19 (82.6)	4 (17.4)	
Interaction with your friends or fellow gamers influences your diet			0.037
Strongly agree	7 (46.7)	8 (53.3)	
Agree	56 (78.9)	15 (21.1)	
Neither agree nor disagree	75 (77.3)	22 (22.7)	
Disagree	59 (79.7)	15 (20.3)	
Strongly disagree	27 (87.1)	4 (12.9)	
Video games influence your diet			0.031 *
Strongly agree	8 (53.3)	7 (46.7)	
Agree	59 (80.2)	14 (19.2)	
Neither agree nor disagree	74 (76.3)	23 (23.7)	
Disagree	60 (76.0)	19 (24.0)	
Strongly disagree	23 (95.8)	1 (4.2)	
Household traditions influence your diet			0.092
Strongly agree	56 (70.0)	24 (30.0)	
Agree	106 (80.9)	25 (19.1)	
Neither agree nor disagree	32 (74.4)	11 (25.6)	
Disagree	17 (81.0)	4 (19.0)	
Strongly disagree	13 (100.0)	0	

* Fisher’s exact test.

## Data Availability

The following data can be downloaded at https://figshare.com/s/de2c45fb9bd7643153d3 (Access from 1 August 2025).
